# Ward round competences in surgery and psychiatry - a comparative multidisciplinary interview study

**DOI:** 10.1186/s12909-019-1554-6

**Published:** 2019-05-08

**Authors:** Elisa Vietz, Esther März, Christian Lottspeich, Teresa Wölfel, Martin R. Fischer, Ralf Schmidmaier

**Affiliations:** 1 Institut für Didaktik und Ausbildungsforschung in der Medizin, Klinikum der LMU München, Ziemssenstrasse 1, 80336 Munich, Germany; 2 Medizinische Klinik und Poliklinik IV, Klinikum der Universität München (LMU), Ziemssenstrasse 1, 80336 Munich, Germany

**Keywords:** Competences, Surgical ward round, Psychiatric ward round, Comparison surgery psychiatry, Entrustable professional activity

## Abstract

**Background:**

The ward round is a key element in everyday hospital inpatient care irrespective of the medical speciality. The underperformance in conducting ward rounds of junior clinicians has already been described. Therefore, necessary skills and competences of clinicians need to be defined, taught and delivered for curricular instruction. In addition to published data on ward round competences in internal medicine this study aims to determine the common competences for surgical and psychiatric ward rounds in order to find differences depending on the speciality.

**Methods:**

Semi-structured interviews with surgical (*N* = 30) and psychiatric ward staff (*N* = 30) of a university hospital and five community hospitals were conducted. Competences necessary for performing ward rounds as well as structural aspects were identified by systematic content analysis and frequency analysis, supported by adequate statistics.

**Results:**

Relevant competences for both fields are: *collaborative clinical reasoning, communication with the patient* and *the team, organization, teamwork, management of difficult situations, self-management, error-management, teaching, empathy, nonverbal communication, patient-management* and *professionalism*. *Clinical skills* were mentioned more often in surgical interviews, while *nonverbal communication* was described more often in psychiatric interviews. *Empathy* and *communication with the team* were more frequently attributed to psychiatric residents.

**Conclusion:**

The competences which were identified as necessary for conducting a ward round in surgery and psychiatry are similar and correspond to previously reported competences in internal medicine. *Clinical skills* are of greater importance in surgery than in psychiatry. Concerning *empathy* and *nonverbal communication*, further research is needed to determine whether they are of minor importance in surgery or whether there is a lack of awareness of these competences.

**Electronic supplementary material:**

The online version of this article (10.1186/s12909-019-1554-6) contains supplementary material, which is available to authorized users.

## Background

Ward rounds are important in every medical field in in-patient care. By definition, hospitalized patients need multidisciplinary care from medical doctors, nurses, therapists, social workers and others - otherwise outpatient care would be appropriate [1]. The traditional ward round brings together information, enables collaborative decision making and provides a platform for communication with the patients and with the team. Studies in internal medicine focus on the ward round, developing checklists [2, 3], analysing participants’ interaction [4] and detecting possibilities for improvement of teaching in ward rounds [5]. But also in other specialities like in surgery and psychiatry, areas of concern regarding ward rounds have been identified. In psychiatry, some patients reported feelings of intimidation [6, 7]. Further concerns were of structural nature, e.g., imprecise appointment times and a large number of participants [7]. In surgery deficits in documentation and in teaching were found [8–10]. Despite these difficulties young clinicians are expected to take an active role in the ward round immediately. A study in internal medicine revealed that final year students may not be sufficiently prepared for conducting a ward round [11]. One way to coach students and young clinicians, while ensuring patient safety at the same time, are simulated ward rounds. Pucher et al. showed that simulation of surgical wards and ward rounds are effective methods to teach patient assessment, management and nontechnical skills [12].

Curriculum development for the teaching of ward rounds requires knowledge about the learning objectives, e.g., regarding ward round competency. Being able to conduct a ward round can hardly be trained as a single skill. It requires different competencies as well as the ability to fulfil several tasks at the same time. One educational method which is suitable for addressing the challenge of teaching and assessing a ward round is the entrustable professional activity (EPA), a concept devised by Ten Cate in 2005 [13]. EPAs represent “tasks or responsibilities that can be entrusted to a trainee once sufficient, specific competence is reached to allow for unsupervised execution” [14]. In an EPA all necessary competences for a certain professional clinical task are linked to the relevant activities. The EPA concept may be employed in conjunction with established competency frameworks, e.g., the framework of the Accreditation Council for Graduate Medical Education (ACGME) or the roles of the Canadian Medical Education Directions for Specialists (CanMEDs) [15–17].

As a lot of prior research about ward round skills has focused on internal medicine, the aim of this study was to identify the competences needed for conducting a surgical and psychiatric ward round. We assumed that these specialities represent quite opposite fields of activity in medical care and therefore exhibit a broad range of medical competences. Our objective was to identify competences relevant in both specialities for conducting a ward round as well as competences more important in one of the two specialities.

## Methods

We chose a qualitative procedure, supplemented with quantitative elements. Interviews about conducting a ward round were performed, evaluated by content analysis and frequency analysis.

### Study sample

The sample contains 30 interviewees for each speciality, surgery and psychiatry, while also including participants working on psychotherapeutic and psychosomatic wards. The interviewees represent experts: clinicians, nursing staff and - for psychiatric and psychosomatic wards - psychologists, all selected for a satisfactory amount of work experience (Table 1). Clinicians in our sample were, on the one hand, senior doctors, i.e. experienced clinicians with completed speciality training, who hold leading positions with responsibility for an organisational unit (ward) in the hospital. These were comparable to consultants or ward attendings (in Germany “Oberärzte”). On the other hand, residents, i.e. clinicians in or with completed speciality training, working under the supervision of a senior doctor. They are comparable to speciality registrars (in Germany “Assistenzärzte”).

**Table 1:** Characteristics of the interviewees

	Resident	Senior doctor	Nursing staff	Psychologists	Total
Surgery					
Number of interviews	12	6	12	–	30
Female	4	1	12	–	17
Average work experience in years	7	22	24	–	
Psychiatry					
Number of interviews	8	6	8	8	30
Female	4	1	5	4	14
Average work experience in years	7	13	25	8	

### Instrument

We used an adapted version of the semi-structured interview guide from a previous study (see Additional file 1), which included open-ended questions as well as direct questions about competences needed for conducting a ward round [18]. Four open-ended questions were aimed at the general ward round process, i.e. questions about the typical ward round, the procedure, the different phases and the subject of the ward round (*procedural part*). Further open-ended questions focused on the tasks of and relevant skills required by a resident and, as interviews hinted at a strong participation of a senior doctor, questions about the tasks and skills of a senior doctor (*tasks-and-skills part*). Direct questions were based on a perusal in internal medicine and were complemented with questions regarding particular topics from the specialities psychiatry and surgery like documentation, empathy and decision making (*literature-based part*) [6–8, 18].

### Implementation of the interviews and systematic text analysis

The feasibility of the interview guideline was assessed in a series of pilot interviews with participants representing the target group. Once practicability of the guideline was reached, one researcher (EV) conducted all interviews. The guideline consisted of both closed and open-ended questions relating to the ward round process and relevant ward round competences and corresponding activities. The interviews were audiotaped and subsequently transcribed with the audio software f4transkript (edu) 2012/ 2013. The transcripts were evaluated by qualitative content analysis using MAXQDA 10 [19]. The coding scheme consisted of precisely outlined definitions of medical competences and their activities with an associated coding agenda. In our analysis we used the main categories of competences, which based on a literature review concerning domains of ward round competences of the previous study [18]. To ensure reliability of codings, six (10%) randomly selected interviews were coded by two raters (EV and EM) with a congruence of 71%.

The analysis focused on the occurrence of competences described by the interviewees in different parts of the interviews.

Firstly, to find common and differing competences between the two specialities we evaluated the frequencies of the competences in the entire interview. Secondly, to find out which competences would be described by the interviewees themselves in the context of the work routine. we evaluated the frequencies of the competences in the procedural part. Finally, to contrast the competences of a senior doctor and a resident we analysed the *tasks-and-skills part*. In summary, the frequencies of the competences described in % were compared applying the following differentiations:

- surgery versus psychiatry
- answers in the *entire interview* versus answers in the *procedural part*
- resident versus senior doctor

For describing the structural aspects, additional noncompetence based codes were taken into account.

### Statistical analysis

To analyse the significance of the relationship between the frequencies of the competences and the medical speciality a chi-squared test with Yates correction (df = 1; defined *p* < .05) and, if required, a Fisher exact test were conducted using Excel 12/ SPSS 22.0. To test an association between the specialities and the competences, the phi coefficient (ɸ) was calculated. To reduce a dependence on the marginal distribution, a corresponding maximum value of phi was computed and phi_norm_ (ɸ_norm_) was calculated. To determine the significance of different obtained frequencies of the competences for a senior doctor and resident, we used the McNemar test, defined *p* < .05.

## Results

A total of 37 h and 26 min of interview material was audiotaped. An interview lasted 32 (SD =8) minutes in surgery and 43 (SD =13) minutes in psychiatry on average.

### Ward round structure

Structural aspects of the ward round described by the interviewees of both specialties are shown in Table 2 and Table 3. Participants from surgery reported that the daily morning ward round is conducted by at least one resident (43%) or a senior doctor accompanied by a resident (57%). Psychiatric ward rounds were described as being led by a senior doctor (87%). However, the senior doctor was sometimes characterized as an irregular participant (7%) or absent (7%). The resident was reported to join the ward round regularly (83%) or intermittently (17%). Most of the interviewees rated the relevance of the ward round in both specialities as high. The estimated duration of the ward round and the time spent with a single patient are shown in Table 3. While surgical ward rounds are described as being conducted frequently but briefly, a large part of psychiatric ward rounds takes place once a week with longer duration.

**Table 2:** Participation in the ward round by role

Participation						
Role	Regular participation [%]		Intermittent participation [%]		No participation [%]	
	Surgery	Psychiatry	Surgery	Psychiatry	Surgery	Psychiatry
Resident	100	83	–	17	–	–
Senior doctor	57	87	–	7	43	7
Nursing staff	90	90	10	7	–	3
Psychologist	–	90	–	–	–	10

Typical ward round participants in percent **[%]** as mentioned by the interviewees in surgical and psychiatric interviews (*N* surgery = 30, *N* psychiatry = 30)

**Table 3:** Temporal aspects of the ward rounds in surgery and psychiatry

Frequency of ward rounds [%]			Estimated duration of ward rounds in minutes		
	Surgery	Psychiatry		Surgery	Psychiatry
Once a week	–	80	Overall duration	43 min	210 min
Twice a week	–	13	Per patient	4 min	12 min
>Twice a week	100	7			

Left column: Described frequency of the ward rounds in percent [%] of the interviews (*N* surgery = 30, *N* psychiatry = 30)

Right column: Reported average duration of the ward rounds in minutes (min)

### Ward round competences in surgery and psychiatry

In the entire interview the following competences were described by over 70% of the interviewees in both specialities: (1) *collaborative clinical reasoning,* (2) *clinician - patient communication,* (3) *clinician - team communication,* (4) *organization,* (5) *teamwork,* (6) *management of difficult situations* and *error-management,* (7) *self-management,* (8) *teaching,* (9) *empathy* and (10) *nonverbal communication* (Table 4). These competences were identified by prior studies about conducting ward rounds (see review of the literature concerning domains of ward round competences [18]) and asked for in the interviews with a direct question.

**Table 4:** Frequency analysis of literature-based competences in the entire interview and in the open-ended questions about the ward round process (procedural part)

Competences	Mentioned by % of the interviewees	
	Entire Interview	Procedural Part
	Surgery - Psychiatry	Surgery - Psychiatry
Collaborative clinical reasoning	100–100	90–93
Communication clinician - patient	100–100	70–83
Communication clinician - team	100–100	83–93
Organization	100–100	57–57
Teamwork	100–100	37–30
Management of difficult situations	100–100	0–3
Self-management	100–100	3–10
Error-management	100–97	0–0
Teaching	97–100	3–0
Empathy	93–100	0–0
Nonverbal communication clinician - patient	70–93^a^	0–0

Frequency analysis of interviews with medical ward staff (*N* surgery = 30, *N* psychiatry = 30) of the specialities surgery and psychiatry

The majority of interviewees of both specialities commented the competences which were identified by literature and asked about in the interviews (first column)

The interviewees described particular competences when answering open-ended questions about the ward round process at the beginning of the interview (second column, procedural part)

^a^Indicates statistically significant differences between surgery and psychiatry: *p* = .045

Additionally, qualitative analysis revealed six further competences, which were not included in the interviews by a direct question, but were mentioned by the experts in the interviews (Table 5). Among these competences, over 67% of the interviewees mentioned (11) *professionalism*, (12) *patient-management* and, for surgery, (13) *clinical skills*.

**Table 5:** Further competences revealed by qualitative interview analysis

Competences	Mentioned in % of the interviews
	Surgery - Psychiatry
Patient-management	93–83
Clinical skills	97–30^a^
Professionalism	67–67
Medical knowledge	30–43
Ability to learn	10–30
Communication clinician - relatives	7–3

Frequency in percent (%) of competences identified by qualitative interview analysis. These competences were not asked for by a direct question in the interview but described by the interviewees

^a^Indicates statistically significant differences between surgery and psychiatry: *p* < .001

### Competences regarding the ward round process

To evaluate which competences would be described by the interviewees themselves as answers to open-ended questions about the work process of the ward round, we analysed the procedural part of the interviews. There, the competences: (1) *collaborative clinical reasoning,* (2) *clinician - patient communication,* (3) *clinician - team communication,* (4) *organization,* and (5) *teamwork* were reported with a frequency of over 30% (Table 4).

### Differing competences in surgery and psychiatry

Statistically significant differences between surgery and psychiatry were found regarding two competences in the entire interview (Table 4 and Table 5): *nonverbal communication between clinician and patient* was more often commented by psychiatric interviewees (S: 70%/ PP: 93%), ɸ = .302, ɸ_norm_ = .636, χ^2^ (1, *N* = 60) = 4.00, *p* = .045 whereas the competence *clinical skills* was described more frequently by surgical participants (S: 97%/ PP: 30%), ɸ = .692, ɸ_norm_ = .909, χ^2^ (1, *N* = 60) = 25.91, *p* < .001.

When contrasting the roles in the ward round of a senior doctor to a resident, significant differences between the specialities surgery and psychiatry appear. In the answers to the questions about the tasks and skills of a resident, codes regarding the competence *empathy* (based on the definition of Mercer and Reynolds [20]) were more often mentioned by the experts as a skill of a psychiatric resident (S: 17%/ PP: 52%), ɸ = .370, ɸ_norm_ = .508, χ2 (1, *N* = 59) = 6.60, *p* = .01. A trend could also be identified for senior doctors (S: 13%/ PP: 44%), ɸ = .330, ɸnorm = .616, χ2 (1, *N* = 43) = 3.33, *p* = .07.

The competence *communication clinician - team* was more frequently described as a competence of a resident in psychiatric than in surgical interviews (S: 50%/ PP: 79%) ɸ = .306, ɸnorm = .419, χ2 (1, *N* = 59) = 4.32, *p* = .04.

### Differences in the competences between the roles of a senior doctor and a resident

**Fig. 1:**
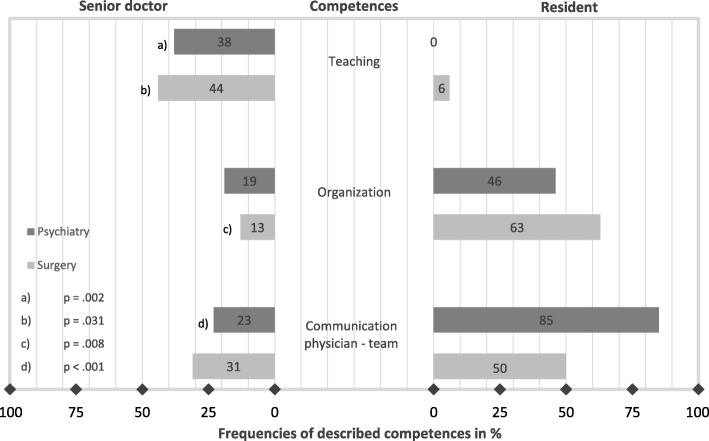
Different attribution of competences to the role of a senior doctor and a resident in surgical and psychiatric interviews. In the context of the required tasks and skills for a ward round, interviewees assigned competences to a senior doctor and a resident. The figure shows only the competences for which significant differences were observed between the roles (*N*_Surgery-Senior_ = 16, *N*_Psychiatry-Senior_ = 26; *N*_Surgery-Resident_ = 16, *N*_Psychiatry-Resident_ = 26))

When contrasting the tasks and skills of a resident with a senior doctor in the ward round, a distribution of competences became apparent (Fig. 1). In both specialities *teaching* was much more frequently described as a competence of a senior doctor with statistically significant differences in surgery, *p* = .031, and psychiatry, *p* = .002. In surgery *organizational competence* was more often assigned to a resident, *p* = .008. A similar trend was identified in psychiatry, this difference did not turn out to be significant, *p* = .065. The competence *communication clinician - team* was, in psychiatry, more often attributed to a resident than to a senior doctor, *p* < .001.

## Discussion

This study shows a comprehensive compilation of ward round competences which cover ward round practice of two rather different disciplines, surgery and psychiatry.

### Ward round competences in surgery and psychiatry

Interviewees of both specialities described the following competences in the entire interview: (1) *collaborative clinical reasoning* (which also includes the diagnostic process and therapy planning)*,* (2) *communication clinician - patient,* (3) *communication clinician - team,* (4) *organization,* (5) *teamwork* (including leadership skills)*,* (6) *management of difficult situations* and *error-management,* (7) *self-management,* (8) *teaching,* (9) *empathy* and (10) *nonverbal communication* (frequency > 70%). These competences were also asked about directly in the interview guide and correspond to findings in the literature concerning ward rounds in internal medicine [18]. The result confirms the relevance of the identified competences and expands them to the domains of the specialities surgery and psychiatry. In addition, the interviewees of both specialities also described competences, which were not asked about in the interviews by direct question. Particularly the competences (11) *professionalism,* (12) *patient-management* and (13) *clinical skills* in surgery were identified to be important (frequency > 67%).

### The focus on individual competences when describing the ward round process

In the open-ended questions about the ward round work process at the beginning of the interview, the experts focus on the competences (1)–(5) among the literature-based competences (1)–(10). This stresses their importance for ward round procedure and they also correspond to skills found in the surgical literature [21, 22]. However, the fact that the other competences (6)–(10) were rarely described spontaneously may also point to a lack of awareness. This is supported by the observation that the experts described them later in the interview, which included direct questions about them.

### Discipline-related differences in ward round competences

We analysed the differences between the specialities surgery and psychiatry regarding the ward round. Structural aspects like duration and frequency of the ward rounds differ strongly. This is a reflection of the differences between the daily routines in both specialities. Nevertheless, the relevance of the ward round was rated to be high by most interviewees in both specialities. Additionally, as described above, many competences required for the ward round are the same in both medical fields.

However, differences in the description of competences were also manifest. The competence *nonverbal communication* was significantly less often described in surgery than in psychiatry, even though the interviews included a direct question about it. Nonverbal behaviour and nonverbal communication play an important role in psychiatry considering the clinician patient relationship and therapeutic aspects [23–25]. However, nonverbal communication is also relevant in other specialities of medical care and has an impact on the final outcome of patient satisfaction [26, 27]. Therefore, further research is necessary to determine whether the results show a deficit in nonverbal communication skills in surgery or whether psychiatric participants need to be more aware of nonverbal actions in the ward round and therefore handle them more consciously than their surgical counterparts.

The competence *clinical skills* is correlated to the surgical speciality. Practical procedures and physical examinations are an important clinical instrument in technical specialities. Patient consultation, for example by checking wounds and drains, is a relevant part in ward round practice and has also been integrated in a ward round safety checklist and in an assessment tool in surgery [21, 28].

For residents a significant correlation of the competence *empathy* to psychiatry was also identified, whereas surgical experts described it with low frequency. This is consistent with the fact that psychiatrists have a higher rating scale in empathy scores than surgeons [29, 30]. Moreover, a psychiatric clinician who is able to empathise during ward rounds can help to improve psychiatric ward round atmosphere, as ward rounds can be an intimidating and, in some cases, frightening situation for patients [6, 7]. Still, when looking at the entire interview, which includes a direct question about empathy, more than 90% of the interviewees in surgery described its role in surgical ward round practice. After all, empathy plays an important role in surgery. Patients, who perceive their surgeons as empathic, have a higher subjective treatment outcome [31, 32]. The everyday surgical ward round with its direct clinician-patient contact may give the surgeon the opportunity to empathise and to establish a well-functioning clinician - patient relationship. The poor description of the competence *empathy* in the open-ended questions in surgery is most likely an indication of poor awareness of it. Therefore self-reflection by using a checklist of ward round competences may help clinicians to increase awareness regarding empathy in ward rounds.

### Role-distribution in ward round practice

Comparing residents to senior doctors with respect to the necessary ward round competences, some differences could be revealed. In contrast to a senior doctor, a resident is expected to focus on the competence *organizatio*n in both specialities, whereas the senior doctor needs to have *teaching* competence. This reveals a role distribution that can ensure patient service as well as educational aspects, particularly when not only the student but also the resident takes the part as a learner. The competence *communication with the team* is significantly correlated to psychiatric residents. It is attributed especially to the resident and not to the senior doctor, as, for example, the resident presents the status of the patient to the senior.

### Competence *teaching* in the ward round setting

Above, it was shown that the competence *teaching* is more likely to be assigned to a senior doctor. It is also noteworthy that the competence *teaching* was only coded once when asking the experts about the general ward round process. This points to the putative opinion that educational aspects play a minor role in the ward round process. Similar effects were identified in studies of Claridge and of Laskaratos et al., in which first year doctors and trainees described the ward round as a suboptimal opportunity for teaching [33, 34]. In a prior study on ward round scripts, it was found that residents do not consider ward rounds as an opportunity for teaching and students see themselves as passive participant of the ward round [35]. EPAs may improve this situation as they represent a useful tool to assess and improve teaching competences - even in senior staff [36]. Therefore, the competence *teaching* ought to be included in the EPA “ward round practice”.

### Teaching of ward rounds - relevance of the identified competences for teaching and assessment

Concerning the teaching of ward round practice itself, it can be noticed that ward round skills and teaching receive more and more attention in surgical literature, as a checklist, an assessment tool and a learning environment for teaching ward rounds have been developed [21, 28, 37]. The surgical assessment tool (SWAT) includes communication, decision making, teamwork, professionalism, situation awareness and leadership as non-technical ward round skills [21]. An interview study about quality markers and improvement measures for surgical ward rounds identified a required skill set for current surgical ward round practice [22]. It includes communication skills, patient assessment/ history taking, diagnostic abilities/knowledge, teamwork/multidisciplinary cooperation, leadership and management skills. Although the semantics of the competences and skills deviates, the contents of the terms are similar and overlap with the competences in our study. In contrast, there are fewer studies about ward round practice in psychiatry. Existing works focus mainly on the patients’ view of ward rounds and practical aspects without studying the required skills of a clinician [6, 7, 38].

While the use of checklists can help graduated clinicians, structured instructions of conducting ward rounds in undergraduate education may give medical students the chance to get ready for the clinical daily routine and may improve ward round practice in general. Nikendei et al. showed that ward round simulation with standardised patients can serve as a valuable learning environment for students [39]. It provides students the opportunity to reflect their performance with the help of peers, tutors or simulated patients [39]. Also, students can train to cope with faults and disruptions in the ward round and to understand the perspective of other ward round participants by playing the role of the patient or nurse [40, 41]. Additionally, students can be taught to organize the ward round, a task that was in this study assigned to residents. Training in organization of the ward round might improve documentation, which is described as a deficit in ward round practice in the literature [8]. Other findings of this study can be critically discussed during ward round training like the role distribution of a senior doctor and resident and the lack of teaching and learning abilities in the actual ward round practice.

An EPA “Conducting a ward round” can provide both, the theoretical background and practical approach for teaching ward rounds in clinical practice or simulation courses [18]. The EPA “Conducting an internal medicine ward round” already represents a detailed framework [18]. As it incorporates the competences of the present work, it can serve as a basis for other specialities.

With our study, we extend prior knowledge on ward rounds in psychiatry and surgery. It provides a sound basis for deriving learning goals as well as for structuring the teaching and learning of ward round practice.

### Limitations

By using a qualitative method supplemented with quantitative elements, this study gives a general overview of the ward round competences in both specialities. For developing an EPA, the general competences discussed in this study would have to be further divided into more specific subcompetences and linked to their observable tasks. While comparing the frequency of the descriptions of the competences, the results have to be interpreted in their context of the respective interview passages (direct question/ open-ended questions) and of the existing literature. Also, statistical analyses have to be seen as supporting the decision which divergent frequencies show a relevant effect. The wards, especially in psychiatry and psychosomatics, also show a marked heterogeneity as the patient groups and therapy concepts can differ. Additionally, the ward rounds depicted in the interviews represent only the status quo of actual ward round practice in Germany, and have to be proven for general validity.

## Conclusions

In summary, the identified competences for conducting a ward round in surgery and psychiatry are similar and correspond to competences in internal medicine, which were found in literature. Relevant competences are: *collaborative clinical reasoning, communication with the patient and the team, organization, teamwork, management of difficult situations and error-management, self-management, teaching, empathy, professionalism* and *patient-management.* Additionally, *clinical skills* were mentioned very frequently in surgery. *Nonverbal communication* is more often described in psychiatry and the competence *empathy* is more frequently depicted as a characteristic of a psychiatric resident than of a surgical one. Experts focus on few competences in the low-structured parts of the interview: *collaborative clinical reasoning, communication clinician - patient, communication clinician - team, teamwork* and *organization.* This stresses the importance of these competences. However, it may also show a lack of awareness regarding other competences.

With this work we have contributed a foundation of competences that can be used to assess and teach ward round practice in both specialities, in surgery as well as psychiatry. A related study, which this work was based on, has proposed an EPA “Conducting an internal medicine ward round” with overlapping competences. This offers the possibility to transfer it to other specialities - bearing in mind the differences.

## Additional file

Additional file 1: Interview schedule. (DOC 84 kb)

## Abbreviation

EPA: entrustable professional activity
