# Supplementary material for: Ward round competences in surgery and psychiatry - a comparative multidisciplinary interview study
Source: BMC Med Educ. 2019 May 8;19:137. doi: 10.1186/s12909-019-1554-6 (PMC6506958; doi:10.1186/s12909-019-1554-6)
Supplement: Supplementary file 1 — Interview schedule. (DOC 84 kb) [file 12909_2019_1554_MOESM1_ESM.doc]

**Interview schedule**

**Description of the study:** We are conducting a study on ward rounds and on relevant skills and abilities required for them. The aim of these interviews is to gain insights on how experienced clinicians and nurses perceive a typical ward round. This information will then be used to establish a concept for a ward round training.

**Handling of data and data privacy:** The collected data will be kept in confidence and personal details will not be passed on. To anonymize data we use a code. For academic analysis and publication of results only the code will be used and data will be presented in a way that no inference to the person of reference is possible.

**Declaration of consent**: Your participation in this study is on a voluntary basis. You may revoke your consent. Do you give your permission in participation in this study (permission is recorded on audiotape)?

**Progress of today’s interview:** First, I will ask you some questions concerning structural aspects of ward rounds. Then, I will ask you questions concerning the relevant skills and abilities needed when conducting a ward round.

*supplemented in psychiatric-psychosomatic interviews

1. Collection of demographic data

| Demographic data | |
| --- | --- |
| Gender | male/female |
| Age |  |
| Profession | clinician; nurse; *psychologist |
| Branch/ Special field |  |
| Wards previously worked at |  |
| Professional position | Resident, Senior doctor |
| Duration of occupation in total/at this hospital (years) |  |
| Number of ward rounds per week |  |

1. Interview

| Understanding of an archetypal ward round | |
| --- | --- |
|  | **What do you consider as a typical ward round on a surgical ward/ * psychiatric/ psychosomatic ward?** |
| The following questions relate to the ward round form which the interviewees described as typical. | |
| Process and Phases of the ward round | |
|  | **Could you please describe the procedure of a typical ward round beginning with the preparation of the ward round?** |
| In case this information was not given, specify with reference to these questions: | At what time does a ward round usually take place?  How often does it take place?  *Is there an arranged appointment time?  *When and how do the patient get the appointment time? |
|  | How long does the ward round typically take? (in total/ per patient)  How do you allot time efficiently during ward round? |
|  | Who is participating in ward rounds? (profession, number of persons)  Is there a maximum number of participants?  *Does the patient have the opportunity not to join the ward round? |
|  | **In which phases can a ward round be subdivided?**  (e.g. before ward round – organization, in front of the door – consultation, in patients room – interaction with the patient, in front of the door – consultation) *(e.g. before the patient is in the room – organisation; patient is in the room – interaction, after the patient left the room – discussion) |
|  | *For how long does the patient attend the ward round? |
|  | In which way does information exchange between the participants take place before, during and after the ward round? |
|  | How does the ward round usually end? How is it generally brought to an end? (e.g. concluding discussion, immediate disbandment)  What is the aim of a ward round? |
| Subject of the ward round | |
|  | **What topics are discussed in the ward round?**  To what extent are issues discussed, which relate to other specialties (e.g. internal medicine)? |
| Tasks and skills | |
| Tasks of a resident and senior doctor | **What function does the resident/ senior doctor have in the course of the ward round?**  **Which specific tasks belong to that function?**  Could you name skills and abilities a resident therefore has to have? |
| Tasks of other participants and interprofessional work | Please briefly portray the tasks of the other participants.  Which role does interprofessionalism play for ward rounds?  What impact does the participation of nurses/ *psychologist have for a ward round? |
| Material and documentation | |
| **Material** | **What kind of materials, documents and tools are needed for the ward round and take with?**  Which records about the patient are needed (charts, records etc.)? Who is responsible for these? |
| **Documentation** | **How is documentation managed on the ward round?**  What is being documented and where? Who documents? What is the relevance of documentation? |
| Team- and self-related aspects | |
| **Decision making** | **Which decisions are usually made during a ward round?**  Who is responsible for making those decisions? |
|  | **How are those decisions made? Is there a decision making process?**  Who takes these decisions?  How is the team involved in decision making?  How is the patient included in decision making?  Are the decision taken before, during or after the patient-contact (in or outside the patients’ room)? |
|  | **Which sources of information are used for decision making?** |
| **Leadership/ Adaption** | **Which challenges is a resident facing when conducting ward rounds?**  Which challenges are posed by leading the team?  How can a resident include the nurse most effectively in the ward round?  Which other persons should be included in the ward round process? Is this actually happening in professional practice?  (If the interviewee can’t find an answer: Think of organisational, personal, team-related or structural challenges.) |
| **Empathy** | **How important is a resident’s empathy with a patient during the ward round?**  If this is classified as not important, why? |
|  | If yes: How do you do this? (If this is easier for you name some examples for situations in which empathy was important.)  Are there situations in which it is not important to empathise with the patient? Are there reasons not to empathise with the patient? |
|  | What do you think, what is the patients’ view on the ward round?  Which aspects make the patient feel comfortable during the ward round? Which aspects make the patient feel uncomfortable during the ward round?  How can one respond to patients, who feel uncomfortable during the ward round? |
| Communication | |
| **Doctor-patient (verbal)** | **How would you describe the doctor-patient communication in the course of the ward round?**  How and to what extent you adapt your language use to the patient and/or the ward round team? (If interviewee can’t answer the question: Could you give an example in which adaption of the language is necessary?)  Could you describe what the adaption looks like?  Which role does the clarification of technical terms have for the patient in the course of the ward round?  How and to what extent does communication impact the doctor-patient relation? |
|  | **How do you determine which information is relevant for the patient?** |
|  | **How do you convey this information?** |
|  | **How far can you assess the resilience of the patient?** |
| **Doctor-patient**  **(non-verbal)** | **How would you describe the non-verbal doctor-patient communication in the course of the ward round?** |
| **Doctor-team** | **How would you describe the communication with the team during the ward round?**  How would you describe the interactions with other participating clinicians, *psychologists?  Is there any interaction between clinician and nurse?  How does the communication between doctor and nurse occur in the course of the ward round?  How important is the communication with the team? |
| **Nurse-patient** | How does communication between patient and nurse happen? |
| Dealing with faults and self-reflection | |
| **Dealing with faults/**  **learning from faults/**  **self-reflection** | **Which difficult situations can occur in the course of the ward round?**  How can difficult situations be handled?  **Which disturbances and interruptions can occur during the ward round?**  How can these disturbances be addressed?  What can the resident do to decrease the effect of these disturbances?  **Which faults can happen in the course of the ward round? How do you address them?** (to the faults he/she is naming)  **When a previously made mistake becomes obvious in the course of the ward round, how would you react?** (e.g. you notice that an examination didn’t take place)  **How do you react when you yourself make a fault in the course of the ward round?** How would you notice it?  Would other team members (e.g. nurses) draw your attention to the fault? If yes, how do you react?  Are there any situations in which you ask others for help (colleagues, team members)? If yes, how would you do it?  **Do the participants reflect the ward round afterwards? How?** |
| Ward round as a teaching environment | |
| **Teaching during ward rounds** | **How much is taught during a typical ward round?**  How much teaching should happen during a typical ward round?  **How could you conduct a ward round to impart knowledge to students?** |
| **Learning to conduct**  **a ward round** | **How did you learn to conduct a ward round?**  When and where? (undergraduate/ postgraduate)  How should future clinicians be prepared for conducting a ward round? Which criteria can be used to decide that a clinician can conduct a ward round autonomously? |
| Relevance of the ward round | |
|  | **How important is the ward round in surgery/ *psychiatry-psychosomatics?** |
| Aspects of a successful ward round | |
|  | **What characterises a successful ward round?**   - **for the clinician** - **for the nurse** - **for the patient** - ***for the psychologist/ for other participants (e.g. social worker)**   How can this be influenced by the clinician? |
| Need to change of the ward round | |
|  | **Is it necessary to change the common ward round practice?** |
| **Closure** | We have talked about different aspects relevant for ward rounds. Is there anything you would like to add? |
|  | Thank you very much for your time! |
